# Usefulness of PIVKA-II for monitoring after liver transplantation in patients with hepatocellular carcinoma

**DOI:** 10.1038/s41598-023-32879-9

**Published:** 2023-04-06

**Authors:** Francisco Villalba-López, Luis Francisco Sáenz-Mateos, Maria Isabel Sánchez-Lorencio, Virginia De La Orden-García, Felipe Alconchel-Gago, Pedro Antonio Cascales-Campos, Carmen García-Bernardo, José Antonio Noguera-Velasco, Alberto Baroja-Mazo, Pablo Ramírez-Romero

**Affiliations:** 1 Murcia’s BioHealth Research Institute, IMIB-Arrixaca, 30120 Murcia, Spain; 2 Division of Clinical Biochemistry, University Hospital Rafael Méndez, 30813 Lorca, Spain; 3 Genetracer Biotech, 28942 Fuenlabrada, Spain; 4 Liver Transplant Unit, University Hospital Virgen de la Arrixaca, 30120 Murcia, Spain; 5 Liver Transplant Unit, Asturias University Hospital, 33011 Oviedo, Spain; 6 Division of Clinical Biochemistry, University Hospital Virgen de la Arrixaca, 30120 Murcia, Spain

**Keywords:** Biochemistry, Cancer, Biomarkers, Gastroenterology, Oncology

## Abstract

The high morbidity and mortality of hepatocellular carcinoma (HCC) has encouraged the search for new biomarkers to be used alongside alpha-foetoprotein (AFP) and imaging tests. The aim of this study was to evaluate the clinical contribution of protein induced by vitamin K absence or antagonist-II (PIVKA-II) for HCC monitoring after liver transplantation (LT) and compare it with AFP, a routinely used tumour marker. A total of 46 HCC patients (Milan criteria) were enrolled in this study. Serum levels of PIVKA-II and AFP were measured before and after transplantation. Clinical features were determined for all the patients that were included. Significant correlations were found between PIVKA-II expression levels and some clinicopathological features, such as tumour size and number of pre-transplant transarterial chemoembolizations (TACEs). Serum levels of PIVKA-II and AFP decreased significantly after LT and increased in patients with tumour recurrence. Serum PIVKA-II levels may play an important role in predicting disease severity. Furthermore, monitoring PIVKA-II levels in HCC transplant recipients reflects the tumor early recurrence after transplantation and could be used, complementing AFP and imaging tests, as a novel biomarker of this pathology.

## Introduction

Hepatocellular carcinoma (HCC) accounts for 75–85% of all neoplasms occurring in the liver. Primary liver cancer was the sixth most commonly diagnosed cancer and the third leading cause of cancer death worldwide in 2020, with approximately 906,000 new cases and 830,000 deaths^1^. Most HCCs occur in patients with underlying liver disease, mostly as a result of hepatitis B or C virus (HBV or HCV) infection or alcohol abuse^2^. Early diagnosis and effective treatment of this disease remains a challenge. If detected early, HCC could be cured with an excellent long-term prognosis, and the main treatment options would be surgical resection or liver transplantation (LT), when the patient is a suitable candidate for transplantation^3^.

Serum alpha-fetoprotein (AFP) has been commonly used as a tumour biomarker for the detection of HCC and to monitor the course of the disease. However this tumour marker has shown a low yield as its values in many cases are normal in early tumours^4^, and 80% of small tumours (under 2 cm) do not express AFP. In addition, patients with liver cirrhosis may have transient elevations of AFP in the absence of HCC^5,6^. The high morbidity and mortality of this disease has encouraged the search for new biomarkers to be used together with AFP and imaging tests, such as positron emission tomography computed tomography (PET/CT), to monitor the evolution of patients after transplantation and predict relapses and metastases^7,8^.

Protein induced by vitamin K absence or antagonist-II (PIVKA-II), also known as des gamma-carboxyprothrombin (DCP), is an abnormal prothrombin molecule resulting from an acquired defect in post-translational carboxylation of the prothrombin precursor in malignant cells^9^ and this marker has been shown to be released in association with vitamin K deficiency and in the presence of HCC^10^. Previous studies on the relationship between serum PIVKA-II level and various clinicopathological factors in HCC have shown that elevated PIVKA-II may be associated with worse tumour behaviour and prognosis in patients with HCC^11^. Serum PIVKA-II level may reflect extrahepatic disease progression after LT better than AFP level, because PIVKA-II can induce tumour cell proliferation and promote tumour angiogenesis^11,12^. Furthermore, PIVKA-II has been recommended as one of the surveillance biomarkers for HCC in at-risk populations and described in the Japanese Society of Hepatology (JSH) guidelines^13,14^. Despite the many studies that have demonstrated the usefulness of PIVKA-II as a marker of HCC and as an important factor in the selection criteria prior to LT, its clinical utility for early detection of HCC recurrence after transplantation remains unclear^12^.

## Methods

### Aim, design and setting of the study

The aim of the present study is to investigate the potential usefulness of PIVKA-II as a biomarker in the follow-up of HCC transplant patients and in the early detection of recurrence in these patients, complementing AFP and imaging tests. This analytical observational prospective dynamic cohort study was conducted at the Virgen de la Arrixaca Hospital (HCUVA, Murcia, Spain) and the Asturias Central Hospital (HUCA). Patients were recruited between September 2014 and May 2021, in two distinct stages: between September 2014 and October 2017 at HCUVA, and between September 2020 and May 2021, at both HCUVA and HUCA.

### Characteristics of participants

The inclusion criteria were HCC patients between 18 and 80 years, candidates for LT and in compliance with the Milan criteria. The diagnosis of HCC was made according to clinical, biological and radiological criteria. It was established based on the presence of at least two compatible imaging scans or the existence of a compatible histological diagnosis. The exclusion criteria were patients with HCC outside the Milan criteria, metastatic HCC and taking vitamin K antagonist drugs, such as the anticoagulants acenocoumarol or warfarin, or vitamin K, in the 6 months prior to sample collection. Most patients underwent at least one session of transarterial chemoembolization (TACE) as bridge therapy until transplantation.

Of the 46 patients recruited after being evaluated by the liver transplant programmes of both hospitals, 35 patients were eventually transplanted and followed up for biomarkers at 1, 6, 12 and 24 months after transplantation. Due to losses in the follow-up of some patients, because of death or any other cause, a total of 46 blood samples were collected pre-transplant, 28 samples at 1 month post-transplant, 24 samples at 6 months post-transplant, 24 samples at 1 year post-transplant and 23 samples at 2 years post-transplant.

### Materials

PIVKA-II was determined by immunoenzymatic assay using chemiluminescence on the LUMIPULSE® G1200 system analyser (Fujirebio Europe N.V., Gent, Belgium), with an analysis range of 5–75,000 mAU/mL and a cut-off value of 48 mAU/mL. Serum AFP levels were analysed by immunochemistry assay on the Cobas 8000 Modular Analyzer series (Roche Diagnostics, Basel, Switzerland) with a measurement range of 0.605–1210 ng/mL and a cut-off value of 11 ng/mL. The number of tumours was determined by histopathological study of the tissue sample obtained during surgery. The remaining clinical variables (aetiology of cirrhosis, tumour size, number of pre-transplant TACEs, vascular invasion, tumour necrosis, presence/absence of recurrence or post-transplant metastasis) were obtained from the clinical history of each patient, considering for tumour size the diameter in centimetres of the largest lesion at diagnosis by imaging test (computerized tomography or magnetic resonance imaging). The presence of vascular invasion and tumor necrosis was determined by liver biopsy of the explant. For the variable aetiology, we distinguished 10 different groups, according to the cause: alcoholism (ALCH), hepatitis B virus infection (HBV), hepatitis C virus infection (HCV), hepatitis B + C virus infection (HBV + HCV), HCV infection plus alcoholism (HCV + ALCH), HBV infection plus alcoholism (HBV + ALCH,) non-alcoholic steatosis (NASH), haemochromatosis (HEMO) and HCV infection + alcoholism + iron overload (HCV + ALCH + Fe). The aetiology of liver disease was considered cryptogenic (CRIPTO) if no cause was identified. Different aetiologies were then classified into 3 groups: viral aetiology (HBV, HCV, HBV + HCV), non-viral aetiology (ALCH, NASH, HEMO, CRIPTO) and mixed aetiology, which encompasses viral plus non-viral aetiology (HCV + ALCH and HCV + ALCH + ALCH + Fe).

### Statistical analysis

The normality of all quantitative variables was tested using the Shapiro–Wilk test. Variables that followed a normal distribution were represented by the mean and standard deviation, while those that did not show a normal distribution were represented by the median and interquartile range (IQR). Qualitative variables were expressed as absolute frequency and relative frequency in percentages. For statistical analysis of the obtained data, Spearman's rho test was used to verify the correlation between biomarkers, as well as between biomarkers and quantitative clinical variables. To study the association between pre-transplant biomarker levels and qualitative clinical variables, the Mann–Whitney U test or the Kruskal–Wallis test was used, depending on whether two groups or more than two groups were compared, respectively. To assess whether there were significant differences in PIVKA-II and AFP levels between the groups before and after transplantation, the Wilcoxon signed-rank test for related samples was performed. Receiver Operating Characteristic (ROC) analysis was performed and the Area Under the Curve (AUC) was estimated to study the predictive efficacy of pre-transplant PIVKA-II and AFP values for post-transplant relapse. The cut-off point for these markers with the best sensitivity and specificity, corresponding to the maximum Youden index, was determined. Multimarker analysis was performed by binary logistic regression by the method of introduction. A pooled ROC analysis was then performed to determine whether additional predictive power could be achieved. IBM^®^ SPSS^®^ Statistics 23.0 software (SPSS, Chicago, Illinois, USA) was used for statistical analysis, considering values of p ≤ 0.05 statistically significant.

### Ethics approval and consent to participate

The study was approved by the Clinical Research Ethics Committee of HCUVA and HUCA hospitals and the subjects gave their written informed consent. All procedures were in accordance with the ethical standards of the institutional and national research committees, as well as with the 1964 Helsinki Declaration and its later amendments.

## Results

The general characteristics of the study population for all patients are shown in Table 1. The median follow-up was 57 months (IQR 4.5–76.5 months), with a minimum follow-up time of 2 months and a maximum follow-up time of 84 months.

**Table 1:** Baseline and pathologic characteristics of 46 patients with HCC on the waiting list for LT. Variables are represented by median and interquartile range or by frequency and percentage, as appropriate.

No. of patients	46
Age (years)	60 (55–63)
No. of tumors	2 (1–3)
Tumor size	2.6 cm (2–4.6)
MELD score	9 (8–12)
No. of preoperative TACEs	1 (0–2)
Sex	n = 46
Male	41 (89.1%)
Female	5 (10.9%)
Etiology	n = 46
Viral (%)	18 (39.13%)
Non-viral (%)	18 (39.13%)
Mixed (viral + non-viral) (%)	10 (21.74%)
Child–Pugh score	n = 32
A	20 (62.5%)
B	9 (28.1%)
C	3 (9.4%)
Tumor size	n = 46
≤ 3 cm	28 (60.9%)
> 3 cm	18 (39.1%)
MELD score	n = 43
≤ 9	24 (55.8%)
> 9	19 (44.2%)
Preoperative TACES	n = 46
Yes	31 (67.4%)
No	15 (32.6%)
Vascular invasion	n = 31
Yes	4 (12.9%)
No	27 (87.1%)
Tumoral necrosis	n = 36
Yes	22 (61.1%)
No	14 (38.9%)
Recurrence	n = 35
Yes	4 (11.43%)
No	31 (88.57%)
Post-transplant death	n = 35
Yes	7 (20%)
No	28 (80%)

*HCC* hepatocellular carcinoma, *MELD* model for end-stage liver disease.

The correlations between PIVKA-II and AFP with clinicopathological variables are shown in Table 2. We observed statistically significant differences in pre-transplant PIVKA-II and AFP values between the group with a diameter of the largest tumour lesion at diagnosis ≤ 3 cm and the group with lesions > 3 cm, such that patients with a tumour size > 3 cm had significantly higher serum PIVKA-II and AFP levels than those with a tumour size < 3 cm. Furthermore, we found statistically significant differences in AFP levels between the different Child–Pugh groups, with patients in the Child C group having significantly higher AFP levels than patients in the Child A and B groups (p = 0.035). Finally, pre-transplant PIVKA-II and AFP levels were not significantly associated with the aetiology of the underlying liver disease, neither when analyzing individual etiologies [PIVKA-II (X^2^ = 15.288; p = 0.083); AFP (X^2^ = 10.441; p = 0.316)] nor when analyzing etiologies in different groups: viral, non-viral or mixed [PIVKA-II (X^2^ = 2.059; p = 0.357); AFP (X^2^ = 2.157; p = 0.340)].

**Table 2:** Correlation between serum biomarkers levels and clinicopathological characteristics using Spearman test.

	PIVKA-II (mAU/mL)	AFP (ng/mL)
Age	0.100 (p = 0.508)	0.135 (p = 0.372)
No. of tumors	0.174 (p = 0.248)	0.206 (p = 0.169)
Tumor size	0.423 (p = **0.003**)	0.189 (p = 0.208)
Number of preoperative TACEs	0.372 (p = **0.011**)	0.130 (p = 0.391)
MELD score	− 0.020 (p = 0.900)	0.075 (p = 0.633)
PIVKA-II (mAU/mL)	–	0.273 (p = 0.067)
AFP (ng/mL)	0.273 (p = 0.067)	–

Significant values are in [bold].

**Figure 1:**
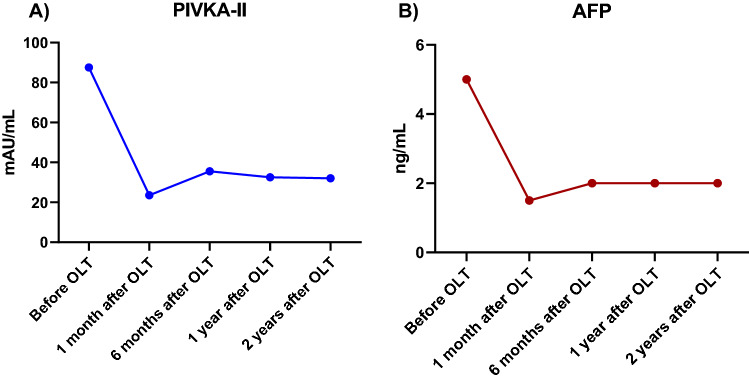
Evolution of median levels of PIVKA-II and AFP after OLT in patients with HCC. *OLT* orthotopic liver transplant.

Table 3 shows the median values of PIVKA-II and AFP at the different times studied, before and after transplantation. In the analysis of the evolution of the biomarkers after transplantation (Fig. 1), PIVKA-II levels experienced a statistically significant decrease at 1 month (Z = − 4.042; p < 0.001), 6 months (Z = − 2.814; p = 0.005) and one year post-transplantation (Z = − 2.315; p = 0.021). AFP levels decreased significantly at 1 month (Z = − 4.262, p < 0.001), 1 year (Z = − 3.257, p = 0.001) and 2 years post-transplant (Z = − 3.436, p = 0.001).

**Table 3:** Median levels of PIVKA-II and AFP in serum of HCC patients before and after transplantation.

Tumor marker	Sample				
	Before OLT	1 month after OLT	6 months after OLT	1 year after OLT	2 years after OLT
PIVKA-II (mAU/mL)					
Median	62	23.50	35.50	32.50	32
IQR	30.75–227.75	14.75–35.75	26.50–37.75	24–41.25	23–53.75
AFP (ng/mL)					
Median	5	1.50	2	2	2
IQR	2.75–6.25	1–2.50	1–2.75	1–3.25	1–5

*OLT* orthotopic liver transplant, *IQR* interquartile range.

Four patients had recurrence, ascertained by imaging, after LT (patients 7, 13, 19 and 23), who eventually died of disease progression. A total of 7 patients died after liver transplantation for various causes. Of the 3 patients who did not relapse, one died after several re-interventions related to biliary stenosis with associated liver abscess, one from a liver abscess leading to septic shock and the last from a cardiovascular event. Pre- and post-transplant PIVKA-II and AFP levels in patients with recurrence are shown in Table 4.

**Table 4:** PIVKA-II and AFP levels in patients with recurrence after transplantation.

Biomarker	Sample	Patient 7	Patient 13	Patient 19	Patient 23
PIVKA-II (mAU/mL)	Before OLT	596	19	725	71
	1 month after OLT	17	112	52	33
	6 months after OLT	271	45	4780	36
	1 year after OLT	–	–	75,000	1915
	2 years after OLT	–	–	–	75,000
AFP (ng/mL)	Before OLT	3	253	11	4
	1 month after OLT	4	9	2	1
	6 months after OLT	93	17	108	2
	1 year after OLT	–	–	163	2
	2 years after OLT	–	–	–	3

*OLT* orthotopic liver transplant.

**Figure 2:**
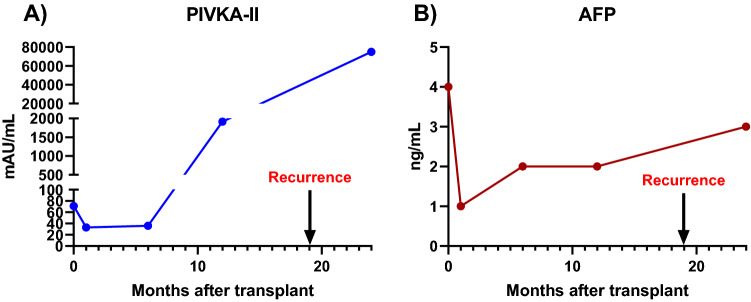
Evolution of PIVKA-II and AFP levels after OLT in patient 23. *OLT* orthotopic liver transplant.

Figure 2 shows an example of variations in tumour marker concentration in a male patient whose age at diagnosis was 47 years and who underwent LT for HCC, the aetiology of which was mixed (HCV and alcoholism). Preoperative PIVKA-II and AFP levels in this patient were 71 mAU/mL and 4 ng/mL, respectively. In this patient, PIVKA-II levels increased sharply at one year post-transplant (1915 mAU/mL) with no elevation of AFP (2.0 ng/mL). AFP levels remained within baseline values at 2 years post-transplant (3 ng/mL) while PIVKA-II increased sharply to 75,000 mAU/mL. This patient was diagnosed with intrahepatic and bone relapse 19 months post-LT.

**Figure 3:**
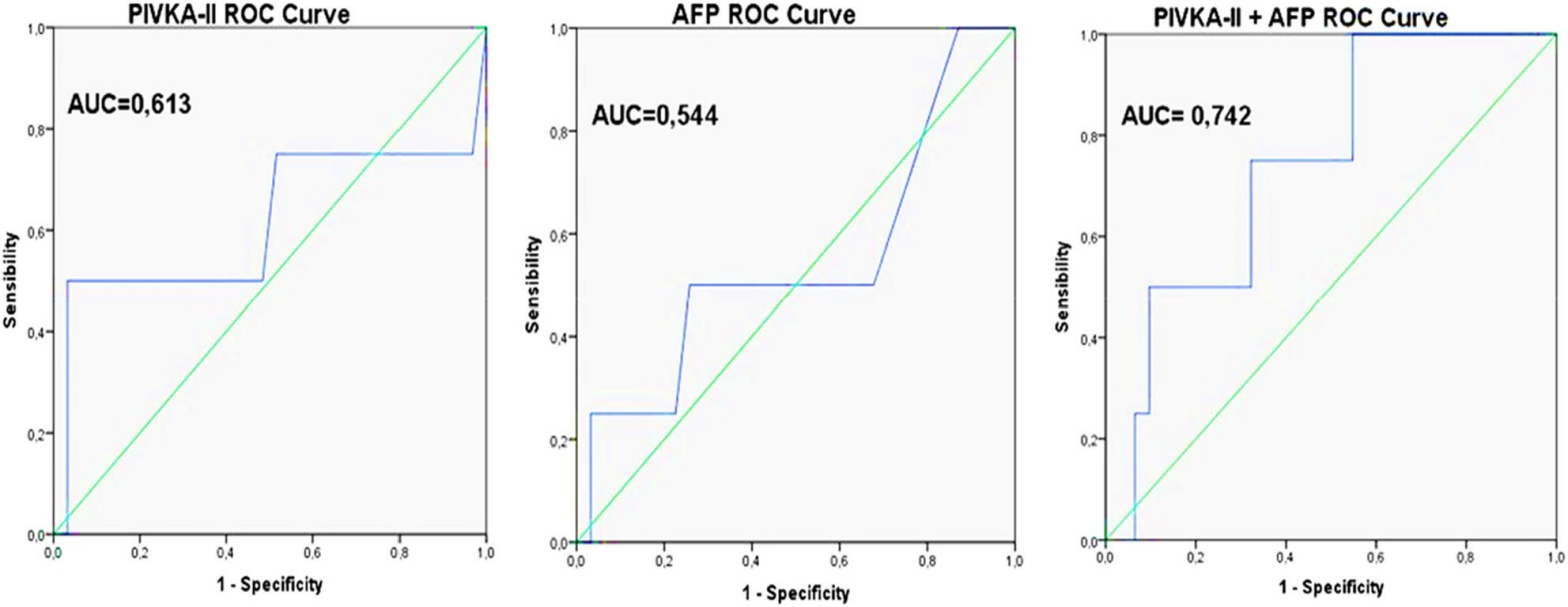
ROC curves of PIVKA-II, AFP and PIVKA-II + AFP.

As shown in Fig. 3, the AUC for determining the post-transplant recurrence/metastasis predictive efficacy of pre-transplant levels of the biomarkers studied was 0.613 (95% CI 0.220–1 and p = 0.468) for PIVKA-II and 0.544 (95% CI 0.206–0.883 and p = 0.776) for AFP. The best PIVKA-II cut-off point for predicting post-transplant recurrence/metastasis with best sensitivity and specificity, corresponding to the maximum Youden index, was established at ≥ 592 mAU/mL, with a sensitivity of 50% and a specificity of 96.8%. For AFP, the best cut-off point for predicting post-transplant recurrence/metastasis with the best sensitivity and specificity was ≥ 9.65 ng/mL, with a sensitivity of 50% and a specificity of 74.2%. After constructing a binary logistic regression model to study the prediction of recurrence of the combination formed by PIVKA-II and AFP and analysing the AUC of this combination of markers using a ROC curve, an AUC of 0.742 (95% CI 0.520–0.964) was found, although these results were not statistically significant (p = 0.120).

## Discussion

HCC is one of the leading causes of cancer-related mortality. Despite strict selection criteria, recurrence occurs in 6–18% transplanted HCC patients^15^. The main reason for this is the presence of residual micrometastases formed even before transplantation, or the spread of tumour cells from the original tumour into the bloodstream during surgical manipulation^16^. Sometimes patients experience extrahepatic metastases even when no primary lesion is found after surgical resection or liver transplantation. In addition, extrahepatic metastases may occur after locoregional therapies for early stage HCC^17^. Serum markers for both early diagnosis of patients at high risk for HCC and early detection of recurrence after transplantation are of great importance as they offer the opportunity to decrease patient mortality and reduce medical costs. Although the role of PIVKA-II has been widely studied in the diagnosis and prognosis of HCC, as well as in the evaluation of surgical treatments such as TACE, there are hardly any studies in the literature that have explored the role of PIVKA-II in the follow-up of patients after LT for HCC.

Regarding the different underlying aetiologies of liver disease in the patients included in our study, HCV is the predominant aetiology, accounting for 34.8% of patients. Overall, taking into account also the combined aetiologies, more than half of the patients had HCV, with this aetiology accounting for 56.5% of the cases. Our results are similar to those of other studies in HCC patients in which HCV is also the predominant aetiology^18–20^. Unlike hepatitis B infection, there is no vaccine for HCV^21^; however, the prevalence of both factors is decreasing due to vaccination of newborns against HBV and the existence of effective treatments for carriers of HBV and HCV^22^. Although some studies have suggested that PIVKA-II and AFP levels are associated with the aetiology of liver disease^23,24^, we did not find significant differences, indicating that the serum levels of the biomarkers analyzed are independent of the etiology of liver disease, in agreement with the findings of Sharman et al.^25^.

When studying the association of pre-transplant PIVKA-II levels with Child–Pugh classification, in our study no statistically significant differences between the different groups were found, so that PIVKA-II levels are independent of the Child–Pugh class to which the patient belongs. Our results are in concordance with those of Saito et al. who also found no significant association between serum PIVKA-II level and Child–Pugh score before treatment with TACE^26^. In contrast, we did find significant differences in serum AFP levels between the different groups of Child–Pugh patients, with Child–Pugh class C patients having significantly higher AFP levels than those in classes A and B, in agreement with previous studies^27^. This finding confirms the ability of AFP to reflect the severity of liver dysfunction in patients with HCC.

After evaluating the association between PIVKA-II and the different clinicopathological parameters of the patients in our study, it was found that patients with larger tumor size had higher levels of PIVKA-II, which suggests that the serum concentration of this biomarker may play an important role in predicting disease severity, such that a higher PIVKA-II concentration may indicate larger tumour volume and worse clinical stage. This correlation has also been observed in other studies in patients diagnosed with HCC, but not on the OLT waiting list^28–31^. PIVKA-II would be a better biomarker for predicting tumor size in HCC than AFP, since although patients with a tumor size > 3 cm had higher serum levels of both biomarkers, AFP did not correlate with tumor size, whereas PIVKA-II did have a statistically significant positive correlation with tumor size. Therefore, we believe that the combination of the levels of both markers would be a better predictor of clinical tumor parameters, such as tumor size, than the determination of only one of them. However, in our study, neither serum PIVKA-II nor AFP levels correlated with the number of tumours, in line with other studies such as that of Feng et al. who found no correlation between serum PIVKA-II levels and the number of tumours^30^, or Lapinski et al. who also found no significant association between serum AFP levels and the number of tumour nodules^32^.

Most of the patients enrolled in the study underwent at least one TACE before liver transplantation. TACE is an effective treatment in patients with unresectable HCC, however most patients treated with TACE will need repeat therapy due to partial response or tumor recurrence. Treatment outcomes after TACE depend on both the severity of underlying liver dysfunction and tumor burden^33^. In our study, pre-transplant PIVKA-II levels correlated with the number of TACEs performed prior to pre-transplant sampling. This result suggests that those patients who received multiple treatments with the intention of reducing the tumor to carry out the transplant had more advanced HCC and a worse clinical stage. Today, the benefits of repeating this treatment are unclear, since on the one hand it does reduce the size of the tumour, but at the same time it could favour tumour spread and local inflammation. Li et al. showed that residual liver cancer cells and normal liver tissue undergo changes in gene expression after TACE, so that the possibility of recurrence and metastasis of residual HCC cells increases after TACE^34^. Furthermore, remnants of normal liver tissue can lead to HCC recurrence, due to cirrhosis and increased compensatory hyperplasia and proliferative activity after TACE. In addition, several previous studies have shown that the establishment of collateral circulation after TACE is an important factor for HCC recurrence and metastasis, which can lead to tumour cell growth with high metastatic potential^35^. Serum AFP levels, in contrast, did not correlate significantly with the number of pre-transplant TACES.

After studying the association between serum PIVKA-II and AFP levels of the patients included in the study, they did not correlate with each other, in agreement with other studies in which, even dealing with HCC patients, they were not candidates for OLT^36–38^. The found results reflect that these two biomarkers are independent of each other, which could be explained by the different synthesis pathways of the two markers in hepatoma cells^39^. Both biomarkers may reflect the tumour burden of HCC patients^40^, and may be complementary markers in terms of their clinical utility^41^.

Ideally, tumour marker levels should fall within a reference range after effective treatment, and rise before imaging studies detect tumour relapse^36^. The exact mechanism by which PIVKA-II is produced by the tumour remains unclear, but the normalisation of PIVKA-II levels after curative treatment of HCC, such as liver resection, clearly indicates that the tumour is the source of its production^42^. In our study, we observed that both PIVKA-II and AFP decreased significantly between pre-transplant and post-transplant values, in agreement with previous studies, such as that of Feng et al., in which serum levels of PIVKA-II and AFP decreased significantly after liver resection^30^. Specifically, in the patients in our study, PIVKA-II shows a statistically significant decrease at 1 month, 6 months and 1 year post-transplantation. In contrast, at 2 years and 3 years post-transplant, we did not find a significant decrease in the levels of this marker. It is important to note that PIVKA-II levels may be elevated in certain situations other than HCC, for example in cases of biliary obstruction^11^. Also the level of PIVKA-II can be strongly influenced by drugs such as rifampicin, vitamin K deficiency (e.g. due to malnutrition in cirrhotic patients) and antivitamin K drugs (e.g. anticoagulants such as acenocoumarol and warfarin)^42^. However, PIVKA-II elevations in patients in our study who did not have post-transplant tumour recurrence were not associated with any specific cause, as none of these patients were found to be on treatment with the aforementioned drugs or to have vitamin K deficiency or obstructive jaundice. AFP levels were significantly lower at 1 month, 1 year and 2 years post-transplantation. Considering that tumour growth patterns are highly variable among individuals, there is probably no single perfect biomarker for HCC monitoring after transplantation; therefore, the combination of biomarkers might be more informative than any single biomarker alone^43^.

Patient 13 was found to have recurrence 6 months after transplantation. PIVKA-II levels in this patient increased at 1 month after OLT, with respect to pretransplant levels, and decreased at 6 months post-transplant, coinciding approximately with the time at which the recurrence was detected by imaging. This patient probably developed tumor recurrence around one month after OLT, so that PIVKA-II would be indicating post-transplant recurrence before imaging tests, such as computed tomography (CT) or magnetic resonance imaging (MRI). However, these levels decreased again at 6 months after transplantation, when HCC recurrence and extrahepatic metastases were detected by imaging, so we cannot affirm that the elevation of PIVKA-II at one month post-transplant was caused by tumor recurrence, even though other causes of PIVKA elevation, already mentioned above, were ruled out, such as the use of anticoagulant antivitamin K drugs. In patients 7 and 19 in our study, the evolution of PIVKA II levels after OLT follows an ideal pattern, in which these levels decreased at 1 month post-transplant with respect to pre-transplant values, reflecting that the diseased liver of HCC patients is the source of production of this abnormal prothrombin molecule, and increased considerably at 6 months post-transplant, coinciding approximately with the detection of tumor recurrence by imaging in both patients. In the case of patient 23, PIVKA-II levels increased abruptly, even before the detection of tumour recurrence by imaging, while AFP levels remained within the reference range. This is in line with the study by Nanashima et al., who showed that changes in PIVKA-II levels after treatment, including hepatectomy and ablation therapy, tended to be more reflective of tumour recurrence than changes in AFP level^44^. Therefore, according to our findings, and despite its limitations, PIVKA-II could be a good marker in the study of progression after liver transplantation, complementing AFP.

After evaluating the predictive ability of pre-transplant AFP and PIVKA-II levels to predict post-transplant recurrence, we found that PIVKA-II was a better predictor of recurrence than AFP, with a PIVKA-II AUC of 0.613 versus AFP AUC of 0.544. Our results are consistent with previous studies^18,45–47^, in which PIVKA-II was also a better predictor of post-transplant recurrence than AFP. This is biologically feasible because PIVKA-II binds to vascular endothelial growth factor receptor 2 and induces autophosphorylation of the receptor and downstream effectors, including phospholipase C-Y and mitogen-activated protein kinase, promoting endothelial cell proliferation and migration. However, AFP is a product of tumour cells and has no biological effect on promoting tumour growth^48^. Combining pre-transplant levels of PIVKA-II and AFP for prediction of post-transplant recurrence increased the AUC to 0.742 with increased sensitivity (100%) but decreased specificity (45.2%). Our results are in partial agreement with other authors such as Chon et al. in whose study sensitivity, but also specificity, were simultaneously improved when both AFP and PIVKA-II levels were measured (sensitivity, 66.7%; specificity, 47.9%) compared to those obtained when only AFP levels were measured (sensitivity 60.1% and specificity 45.2%) or PIVKA-II levels alone (sensitivity 62.9% and specificity 47.9%)^49^. Lee et al. also demonstrated that the combination of AFP and PIVKA-II was a better predictor of recurrence after transplantation in advanced HCC than either marker alone^50^. Thus, this combination of markers could improve the ability of each marker alone to identify patients most likely to have post-transplant recurrence, although the results were also not statistically significant (p > 0.05). The number of patients was a limitation of these studies due to two factors: on the one hand, patients must meet a series of strict requirements to be on the waiting list and be candidates for transplantation, and in addition, there must be a donor with an organ compatible with the recipient. The other limitation is the moderate post-transplant recurrence rate of 6–18%, both of which contribute to severely limit the cases under study. In this context, the results of this study may be useful for the development of new lines of research that provide new evidence for PIVKA-II to be incorporated as a useful biomarker in the management of HCC in the clinical laboratory, as occurs in countries such as Japan, where together with ultrasound and *lens culinaris* agglutinin-reactive fraction of AFP (AFP-L3), it is used in the screening of at-risk patients every 6 months^51^.

## Conclusions

Pre-transplant PIVKA-II concentration in peripheral blood in patients with HCC is significantly associated with clinical factors such as larger tumour size and the number of TACEs performed before transplantation, however the levels of this biomarker are independent of the aetiology of the underlying liver disease. According to the results of the present study, high levels of this tumour marker could play an important role in predicting disease severity by indicating larger tumour volume and worse clinical stage.

Monitoring PIVKA-II levels in HCC transplant recipients reflects the tumor early recurrence after transplantation and could be used, complementing AFP and imaging tests, as a novel biomarker of this pathology. Despite not reaching statistical significance, pre-transplant levels of PIVKA-II are better predictors of post-transplant recurrence than AFP, and the use of these biomarkers together may improve the predictive ability of using AFP alone. Althogh PIVKA-II is already included in clinical practice guidelines in some Asian countries^52–54^, there are practically no published studies on the role of PIVKA-II in the follow-up of HCC after liver transplantation in Western countries. Our findings place PIVKA-II as a promising worldwide marker, serving as a basis for future large-scale studies or meta-analyses that can strengthen its usefulness in clinical practice.

## Data Availability

The datasets used and/or analysed during the current study are available from the corresponding author on reasonable request.

## Abbreviations

AFP: Alpha-fetoprotein
AFP-L3: *lens culinaris* Agglutinin-reactive fraction of AFP
ALCH: Alcoholism
AUC: Area under the curve
CRIPTO: Cryptogenic
CT: Computed tomography
DCP: Des gamma-carboxyprothrombin
Fe: Iron overload
HBV: Hepatitis B virus
HCC: Hepatocellular carcinoma
HCUVA: Virgen de la arrixaca hospital
HCV: Hepatitis C virus infection
HEMO: Haemochromatosis
HUCA: Asturias Central Hospital
IQR: Interquartile range
JSH: Japanese Society of Hepatology
LT: Liver transplantation
MELD: Model for end-stage liver disease
MRI: Magnetic resonance imaging
NASH: Non-alcoholic steatosis
OLT: Orthotopic liver transplant
PET/CT: Positron emission tomography computed tomography
PIVKA-II: Protein induced by vitamin K absence or antagonist-II
ROC: Receiver operating characteristic
TACE: Transarterial chemoembolization
